# Interaction Effect of Sex and Body Mass Index on Gray Matter Volume

**DOI:** 10.3389/fnhum.2019.00360

**Published:** 2019-10-17

**Authors:** Yufei Huang, Xianjie Li, Todd Jackson, Shuaiyu Chen, Jie Meng, Jiang Qiu, Hong Chen

**Affiliations:** ^1^Faculty of Psychology, Southwest University, Chongqing, China; ^2^Department of Psychology, Faculty of Social Sciences, University of Macau, Taipa, China; ^3^Key Laboratory of Cognition and Personality, Southwest University, Ministry of Education, Chongqing, China

**Keywords:** body mass index (BMI), voxel-based morphometry (VBM), gray matter volume (GMV), sex interaction, resting state functional connectivity (RsFC)

## Abstract

**Objective**: Few studies have investigated sex differences in brain structure associated with body mass index (BMI), and the related findings are inconsistent. In this study, we aimed to investigate the effect of sex × BMI interactions on gray matter volume (GMV), and to determine the implications of any structural differences.

**Methods**: The final sample comprised 653 participants (449 women) who were assessed using voxel-based morphology analysis of T1-weighted magnetic resonance images. We used the voxel-based morphometry (VBM) to build a multiple regression model to explore the association between BMI and GMV, and used analysis of variance (ANOVA) to explore the BMI × sex interaction on GMV. A subset of 410 participants (291 women) underwent whole brain resting-state functional connectivity (rsFC) analysis to investigate sex differences in the seed (interaction) region. The cluster with a significant effect in the previous ANOVA analysis was used as a seed.

**Results**: A significant BMI × sex interaction was observed in the left anterior cingulate cortex (ACC), while GMV was negatively correlated with BMI in men but not in women. The rsFC between the left ACC and the caudate was lower in men than in women. Within the entire sample, the insula, caudate, and medial frontal cortex activities were negatively correlated with BMI while the cerebellum and postcentral gyrus activities were positively correlated with BMI.

**Conclusions**: Our findings address the interaction effect of BMI and sex on GM alterations. We found that the GMV in men seemed to be more likely to change with BMI than women, and the left ACC may be the reason for the increase in BMI of men, but not women.

## Introduction

There are potential sex differences in the associations between body mass index (BMI) and regional gray matter volume (GMV) in the human brain. While numerous morphometric studies have investigated obesity-related alterations in brain structure, typically, sex has been treated as a covariate for which researchers have exhibited little interest (He et al., 2015; Willette and Kapogiannis, 2015).

Research on whether there are sex differences in the relationship between BMI and brain structures has yielded conflicting results. Some studies have found significant sex differences, while some have not. Moreover, in the studies which found sex differences, the brain regions with significant results were also different. For example, one study conducted by Taki et al. (2008) in a large Japanese cohort suggests that there may be interactions between sex and BMI on obesity-related structural brain changes. The gray matter (GM) ratio (which was defined as the percentage of the GMV divided by the intracranial volume, to normalize differences in head size) was negatively correlated with BMI in men but not in women. Moreover, a voxel-based morphometry (VBM) study indicated that the GMV of some brain regions in men was positively (the bilateral inferior frontal gyri, posterior lobe of the cerebellum, frontal lobes, temporal lobes, thalami, and caudate heads) or negatively (the bilateral medial temporal lobes, anterior lobe of the cerebellum, occipital lobe, frontal lobe, precuneus, and midbrain) correlated with BMI, but not in women. In another study, Horstmann et al. (2011) found a significant correlation between the GMV in the putamen and BMI, and a negative correlation between leptin levels and the GMV in the right dorsolateral prefrontal cortex (dPFC) in women only.

However, other studies identified a relationship between BMI and brain structure but did not observe sex differences (Raji et al., 2010; Karlsson et al., 2013; Veit et al., 2014). For example, Veit did not find sex differences in cortical thickness as a function of BMI or visceral fat (Veit et al., 2014). Karlsson et al. (2013) found that BMI-related white matter (WM) atrophy was similar in both sexes. However, these findings were based on relatively small sample sizes, which limited the statistical power.

In most countries, the obesity rate and BMI are higher in women than in men (Power and Schulkin, 2008). Moreover, the body areas where women and men tend to accumulate fat are different (Lovejoy et al., 2009). Previous studies have demonstrated that abdominal fat is more harmful to the cardiovascular system than is subcutaneous fat (de Koning et al., 2007). Kurth et al. (2013) found that GM reduction related to weight status is more widespread when measured by waist circumference than BMI. Thus, the reduction in GM relative to weight gain may differ between men and women.

A study with a sample of young adults in China revealed significant negative correlations between BMI and GMV in the midcingulate cortex (MCC), left orbitofrontal cortex (OFC), and left ventromedial prefrontal cortex (vmPFC; He et al., 2015). However, the researchers did not consider potential sex differences in the relationship between GMV and BMI. Therefore, we aimed to clarify whether there is a sex × BMI interaction effect on GMV.

## Materials and Methods

### Participants

The participants were young adult undergraduate students recruited *via* online advertising and leaflets distributed on campus for research on associations between the brain, emotion, cognition, and personality. The study was conducted in the Key Laboratory of Cognition and Personality at Southwest University (SWU), Chongqing (for more details please visit http://www.qiujlab.com) and was approved by the Institutional Review Board of the SWU Imaging Center for Brain Research. Written informed consent was obtained from all participants.

Individuals who are not able to undergo magnetic resonance imaging (MRI) were excluded from this study (e.g., metal material in the body, claustrophobia, etc.). Participants who reported a history of neurological or psychiatric disease, and/or substance abuse, or those who exhibited excessive scanner artifacts were excluded from this study (19 participants were excluded because their image quality was poor, and 37 participants were excluded due to a lack of critical information such as sex or weight). In total, 653 volunteers (449 women) underwent a T1-weighted MRI scan during the 1.5-years period of study. Prior to scanning, participants were weighed on an electronic scale and their height was measured by an experimenter using a measuring tape fixed to a wall. Participants’ BMIs were calculated as weight divided by height squared (kg/m^2^). The resting-state functional MRI (Rs-fMRI) scans of 410 participants (291 women) were also included in the analysis. The (Rs) data of the remaining participants were either not collected (*n* = 219) or lost due to excessive head movements (*n* = 24).

### MRI Data Acquisition

All MRI data were acquired with a 3.0-T Siemens Trio scanner (Siemens Medical, Erlangen, Germany). High-resolution 3-dimensional T1-weighted anatomical images were acquired using a magnetization-prepared rapid gradient echo sequence [repetition time (TR) = 1,900 ms, echo time (TE) = 2.52 ms, inversion time = 900 ms; slices = 176; slice thickness = 1.0 mm; flip angle = 9°; resolution matrix = 256 × 256; voxel size = 1 × 1 × 1 mm]. During Rs-fMRI scanning (duration, 8 min), participants were asked to keep their eyes open without thinking about anything in particular. Rs functional images were acquired using gradient echo planar imaging sequences, with the following parameters: slices = 32; flip angle = 90°; TR/TE = 2,000/30 ms; field of view = 220 × 220 mm; slice gap = 1 mm; thickness slice = 3 mm; voxel size = 3 × 3 × 4 mm^3^.

### MRI Data Preprocessing

VBM analysis was performed with SPM8 (Welcome Trust Center for Neuroimaging, London, UK^1^) framed in Matlab2014 (Math-Works, Natick, MA, USA). Each image was first checked for artifacts and then manually re-oriented to the anterior commissure-posterior commissure line for better registration. Images were then segmented into WM, GM, and cerebrospinal fluid using a unified segmentation approach (Ashburner and Friston, 2005). The DARTEL registration method was used to produce custom templates (GM, WM) from the entire sample (Ashburner, 2007). Each participant’s images were normalized into the Montreal Neurological Institute (MNI) space, modulated, and smoothed with an 8 mm full width at half maximum (FWHM) Gaussian kernel to increase the signal-to-noise ratio. Absolute masking with a threshold of 0.2 was applied.

### Rs Image Preprocessing

Data processing assistant for resting-state software and REST toolkit7 were used to process the Rs data (Yan and Zang, 2010; Song et al., 2011). Both toolboxes were employed within the MATLAB2014 framework. According to the standard protocol suggested by Friston et al. (1996), the first ten imaging volumes were dismissed. We also used the proposed parameters of Yan, regarding slicing time and head motion corrections of the Friston 24 parameter (Friston et al., 1996), in addition to spatial normalization to a standard template, resampling to 3 mm^3^ voxels, smoothing with a 6 mm FWHM kernel, linear detrending, and filtering with a band pass filter (0.01–0.08 Hz). Specific covariates (e.g., global signal, WM, and cerebrospinal fluid) were regressed to reduce noise.

### Statistical Analysis

#### Whole-Brain VBM

VBM was performed using SPM8. We used a multiple regression model to explore the association between BMI and GMV. The total GMV, sex, and age were set as covariates for the whole-sample analysis. Other options in the factorial design interface were set to the default mode. Multiple comparisons on peak intensity were family-wise error (FWE)-corrected (*P* < 0.05). SPM images were projected to an MNI template. The BMI × sex interaction effect on GMV was tested *via* a two-way analysis of variance (ANOVA) with sex as between factor, BMI as within-subjects factor, age and total GMV as covariates.

#### Seed-Based Whole-Brain Voxel-Wise RsFC

Seed-based whole-brain voxel-wise resting state functional connectivity (RsFC) analysis was performed using SPM8. In order to determine whether areas exhibiting structural differences also differed in functional connections, clusters maintained in the sex-specific VBM analysis were re-sliced as seed regions (i.e., regions of interest). We saved the significant regions in the ANOVA as a mask file, then add the file as seed in the RsFC analyses. Maps from seed to voxel-wise whole brain RsFC analyses were produced. We then analyzed sex differences in whole-brain RsFC seeds, using age and BMI as covariates. Significance was set at *P* < 0.05, and multiple comparisons on intensity level were FWE-corrected (*P* < 0.05, voxel level).

## Results

### Sample Characteristics

The mean BMI of the sample was 20.69 [standard deviation (SD) = 2.61], with an average age of 19.51 years (SD = 1.51). The BMI ranged from 15.4 to 34. The BMI of 107 participants (62 women, 45 men) was not <23, and 16 of them (six women, 10 men) had a BMI of >27. As indicated in Table 1, sex differences were observed in BMI, which was slightly higher for men than women.

**Table 1:** Sample characteristics for voxel-based morphometry (VBM) and resting state of our participants.

Data	Measure	Men	Women	*t*	*p*
T1 image	Age (years)	19.54 (1.53)	19.49 (1.49)	0.370	0.712
	Height (cm)	172.50 (5.77)	160.69 (5.66)	24.558	<0.001
	Weight (kg)	62.91 (9.84)	52.95 (7.14)	12.993	<0.001
	BMI (kg/m2)	21.13 (3.02)	20.49 (2.38)	2.649	0.008
	N	204	449		
Resting-state	Age (years)	19.55 (1.65)	19.59 (1.69)	−0.246	0.806
	Height (cm)	171.59 (5.55)	160.59 (5.61)	18.056	<0.001
	Weight (kg)	60.96 (9.88)	53.05 (7.29)	7.899	<0.001
	BMI (kg/m2)	20.67 (2.89)	20.55 (2.43)	0.421	0.674
	N	119	291		

*All the numbers in the form of N(M) represent mean and standard deviation. The numbers in the parenthesis refer to the standard deviation; the numbers before the parenthesis refer to the mean value*.

### VBM Results

**Figure 1:**
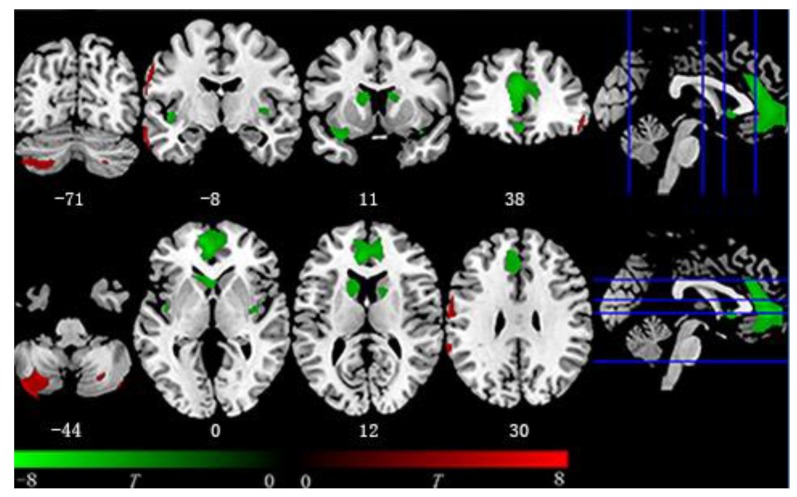
Voxel-based morphometry (VBM) result. Regions covered by green color are negatively associated with body mass index (BMI) while red color represents the opposite. Negative region including bilateral insula, bilateral caudate, and medial frontal region from orbitofrontal cortex (OFC) to midcingulate cortex (MCC). Positive region including bilateral cerebellum and postcentral gyrus.

Within the entire sample, after FWE correction, the multiple regression model revealed that BMI was negatively associated with GMV in the following brain regions: the bilateral insula, bilateral caudate, right middle cingulate gyrus, and a large cluster in the medial frontal cortex, extending from the OFC to the anterior cingulate cortex (ACC). BMI also had positive correlations with GMV in the bilateral middle temporal gyrus, bilateral posterior cerebellum, right inferior frontal gyrus, left postcentral gyrus, and supramarginal gyrus (Table 2 and Figure 1).

**Table 2:** VBM analyses of relationships between BMI and GMV: whole sample.

Results	Voxels	*T*	Brain regions	*X*	*Y*	*Z*
Positive	1,020	6.27	Cerebellum Posterior Lobe-L	−30	−64	−45
	265	5.87	Middle Temporal Gyrus-L	−68	−9	−18
	51	5.68	Middle Temporal Gyrus-R	62	4	−29
	122	5.62	Inferior Frontal Gyrus-R	52	38	−9
	76	5.19	Cerebellum Posterior Lobe-R	27	−67	−44
	95	5.16	Supra Marginal-L	−64	−42	28
	297	5.06	Postcentral Gyrus-L	−63	−2	28
	22	4.94	Cerebellum Posterior Lobe-R	34	−81	−51
	13	4.75	Inferior Frontal Gyrus-L	−51	41	−6
	14	4.63	Postcentral Gyrus-R	46	−34	52
Negative	4,076	6.60	Bilateral ACC, MCC, OFC, medial frontal gyrus	−12	56	−2
	814	5.77	Caudate-L, Extra-Nuclear-L, Putamen-L	−4	21	−2
	544	5.75	Insula-R, Inferior Frontal Gyrus-L	−33	12	−20
	25	5.48	Parahippocampa Gyrus-L	−14	2	−20
	281	5.28	Caudate-R, Extra-Nuclear-R, Putamen-R	15	3	16
	67	5.27	Insula-R	38	14	−18
	100	5.00	Superior Frontal Gyrus-L	−6	68	−9
	176	4.96	Insula-R, Putamen-R, Extra-Nuclear-R	34	−9	1
	39	4.76	Middle Cingulate Gyrus-R	8	−16	39
	14	4.69	Middle Cingulate Gyrus-L	−8	−22	34

*X, Y, Z represent MNI coordinate; R and L separately represent the right hemisphere and the left hemisphere; voxel size is 1.5 × 1.5 × 1.5 mm^3^. ACC, anterior cingulate cortex; MCC, middle frontal cortex; OFC, orbital frontal cortex*.

**Figure 2:**
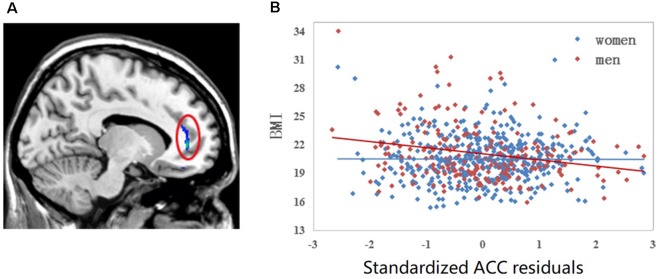
Linear regression of BMI to left anterior cingulate cortex (ACC) volume. **(A)** Colorful cluster in the red ellipse is located on the left ACC where we detected significant sex by BMI interaction effect family-wise error (FWE; *P* < 0.05, voxel level). **(B)** Scatter plot of left ACC is shown. The correlation between BMI and the gray matter volume (GMV) of left ACC is different between men and women after controlling for the whole GMV and age. For men sample, *r* = −0.229, *P* = 0.001; for women sample, *r* = −0.004, *P* = 0.933.

The results of the two-way ANOVA revealed a significant interaction effect (FWE, *P* < 0.05, voxel level) of BMI × sex in the left ACC where GMV was negatively correlated with BMI among men but not women. A scatter plot of this interaction effect is provided in Figure 2.

### Seed-Based Whole-Brain Voxel-Wise Resting State Functional Connectivity

After FWE correction (*P* < 0.05, voxel level), the cluster with a significant effect in the previous ANOVA analysis was used as a seed. Only one cluster in the left ACC survived after multiple comparison correction. The RsFC of seed regions and the left ACC was significantly higher among women than men (FWE *P* < 0.05, voxel level, Table 3).

**Table 3:** Sex differences on seeds to whole brain voxel-wise resting-state functional connectivity (RsFC) analyses.

Seed	Voxels	*T*	*X*	*Y*	*Z*	Brain regions
***Women > Men***						
Left ACC	76	7.43	−9	15	12	Left Caudate
	31	6.52	9	12	12	Right Caudate
	11	5.62	−12	72	−3	Left Medial Frontal Cortex
***Men > Women***						
						none

*X, Y, Z represent MNI coordinate; voxel size is 3 × 3 × 3 mm^3^. ACC, anterior cingulate cortex*.

## Discussion

Our study found that there is a sex difference in the relationship between BMI and GMV. Specifically, we found that the GMV of the left ACC was negatively correlated with BMI in men but not in women. This difference is consistent with a previous finding of a negative association between GM and BMI in men, but not in women (Taki et al., 2008). Leptin may be a potential factor associated with the GMV reductions in the ACC. For example, in a study of three patients with hereditary leptin deficiencies, the ACC GMV increased following leptin replacement (Matochik et al., 2005). Moreover, women typically have higher serum leptin levels than do men, as women hormones may increase leptin responsiveness (e.g., leptin transport, leptin receptor expressions) in the brain (Lovejoy et al., 2009). In general, men’s GMV may be more likely to change as BMI increases, and we provide new evidence for this. Moreover, the differences we found on the left ACC may be related to leptin. Future studies can explore whether there is a sex difference in relationships among BMI, GMV and leptin level.

The left ACC region in which men exhibited decreased GMV with elevations in BMI may reflect the FC results. The rsFC between the left ACC and the left caudate, both of which are dopamine projection regions with possible involvement in motor control and reward (Björklund and Dunnett, 2007), was lower in men than in women. Two previous studies reported that obese individuals have higher FC between the vmPFC and the ventral caudate (Coveleskie et al., 2015; Contreras-Rodríguez et al., 2017). Cortico-striatal connectivity is thought to play a key role in goal-directed behaviors. Combined with the previous finding, which shows that the GMV of the left ACC was negatively correlated with BMI in men but not in women, our RsFC results suggest that the structure and function of the left ACC may be the reason for the increase in BMI of men, but not women.

Consistent with previous studies, the GMV had negative associations with BMI in a wide range of brain regions, including the caudate nucleus, insula, putamen, middle and anterior cingulate cortices, medial frontal cortex, and OFC within a large young adult sample. These brain areas have been implicated in processing reward, hedonic experiences, interoceptive awareness, taste, and habitual behaviors (for example, see Hinton et al., 2004; Yau et al., 2014; Tuulari et al., 2016).

The largest cluster to have a negative correlation with BMI was located in the medial frontal lobule and extended from the rectus to the ACC. This brain region has been consistently reported in studies exploring the associations between GM alterations and obesity (Raji et al., 2010; Walther et al., 2010; Hassenstab et al., 2012; Tuulari et al., 2016). The OFC has been proposed to mediate hedonic value, as well as food salience (Kringelbach et al., 2003; Hinton et al., 2004). In a previous study on older women, reduced OFC volume was associated with poor executive function (Walther et al., 2010). Moreover, disinhibited eating of obese adolescents was related to reduced OFC volume and executive dysfunction (Maayan et al., 2011). Other studies have linked significantly lower cortical thickness of the ACC with an increased likelihood of obesity (Hassenstab et al., 2012; Yau et al., 2014). Our sample was similar to that of He et al. (2015) who also assessed college students with a relatively low mean BMI. In line with their results, the middle cingulate cortex was negatively correlated with BMI in our sample (He et al., 2015). Further, the ACC and MCC appear to be involved in impulse control (Cole et al., 2009; Shackman et al., 2011). In agreement with previous findings, our results indicate that GMV reductions in brain structures related to reward, decision making, and impulse control are more likely to be observed in young individuals with relatively high BMIs.

Reassuringly, the negative association between GMV in the bilateral dorsal striatum (putamen and caudate) and BMI replicates the results of studies from other countries, strengthening the line of evidence that dorsal striatum attenuations are related to accumulation of adipose tissue (Pannacciulli et al., 2006; Kurth et al., 2013). In terms of function, previous studies have implicated dorsal striatum involvement in food-related reward (Kringelbach et al., 2003). For example, Small et al. (2003) demonstrated that activation of the dorsal striatum was associated with subjective pleasantness during food consumption. Moreover, a recent longitudinal study on adolescents revealed that GM loss in the putamen was associated with increased body fat (Yokum and Stice, 2017). RsFC between the dorsal striatum and somatosensory cortex is also correlated with food cravings and future weight gain (Contreras-Rodríguez et al., 2017).

We also observed positive associations between GMV and BMI. For example, BMI elevations corresponded to higher GMV in the cerebellum, middle temporal gyrus, and postcentral gyrus. These findings align with those of previous reports (Pannacciulli et al., 2006; Taki et al., 2008) but there is no clear explanation for these correlations. Our finding is in line with previous reports of GMV positively correlating with BMI, but these studies lacked evidence to explain the mechanism underlying the relationship between BMI and GMV.

This study has notable strengths including a comparatively large sample with sufficient statistical power to detect subtle effects and a focus on examining sex differences in the associations between BMI and brain structures and connectivity. Our findings provide a solid foundation for further hypotheses tests of the risk of increased BMI in men. Nonetheless, the main limitations of this work also warrant consideration. First, there are fewer overweight or obese individuals in our sample. Our study found sex differences in the relationship between BMI and GMV, which the BMI of men with less GMV in left ACC is more likely to increase. But due to the limitation of the BMI of our sample, whether the left ACC is a key brain region for overweight and obesity in men but not in women is still unknown. Second, our sample was limited to young adults in college, thus the results do not necessarily extend to other populations. For example, given that menopause may have a substantial impact on BMI-related GM alterations (Soreca et al., 2009), it is not clear whether the same pattern of results would be observed among middle-aged adults. Third, BMI is a conventional index used to assess weight status but only estimates body fat indirectly. Other measurements (e.g., waist circumference, body fat rate) should also be considered in future studies. Finally, the use of a non-experimental, cross-sectional design is a limitation with respect to elucidating BMI as a cause vs. an effect in GMV studies. However, in light of the current correlational results, future experimental studies in animals and longitudinal studies in humans may elucidate the effects of BMI increases on GMV changes.

## Conclusion

In summary, our study revealed reduced GMVs in relatively young individuals with high BMIs. Further, we found that the GMV in men seemed to be more likely to change with BMI than women. Our findings are significant since they address the interaction effect of BMI and sex on GM alterations, which has often been neglected in previous studies. In addition, understanding how GMV changes with increasing BMI, and the sex differences in BMI and GMV in a normal sample can also help develop more appropriate and targeted prevention of obesity and overweight.

## Data Availability Statement

The datasets generated for this study are available on request to the corresponding author.

## Ethics Statement

The studies involving human participants were reviewed and approved by the Institutional Review Board of the SWU Imaging Center for Brain Research. The patients/participants provided their written informed consent to participate in this study.

## Author Contributions

YH: writing and modifying. XL: analyzing and writing. YH and XL: contributed equally to this work. TJ: modifying. JM: analyzing. SC: analyzing. JQ and HC: design and modifying.

## Conflict of Interest

The authors declare that the research was conducted in the absence of any commercial or financial relationships that could be construed as a potential conflict of interest.

## Footnotes

^1^https://www.fil.ion.ucl.ac.uk/spm/software/spm8/

## Abbreviations

BMI: Body mass index
GMV: gray matter volume
GM: gray matter
WM: white matter
VBM: voxel-based morphometry
MCC: midcingulate cortex
OFC: orbitofrontal cortex
FWE: family-wise error
ACC: anterior cingulate cortex
RsFC: resting state functional connectivity
SWU: Southwest University
MNI: Montreal Neurological Institute
FWHM: full width at half maximum
fMRI: functional MRI.
